# Supplementary material for: In silico drug repurposing at the cytoplasmic surface of human aquaporin 1
Source: PLoS One. 2025 Jan 9;20(1):e0314151. doi: 10.1371/journal.pone.0314151 (PMC11717375; doi:10.1371/journal.pone.0314151)
Supplement: S1 Table — (PDF) [file pone.0314151.s001.pdf]

Supplementary data

Table 1: Compounds excluded from MD study at mid-study review and the reason for exclusion.

Compound	Reason for exclusion
**Acebutolol**	Infrequently prescribed
**Dobutamine**	Similar to other compounds tested
**Triethylenetetramine**	Infrequently prescribed
**Azelaic acid**	Infrequently prescribed
**Glucuronic acid**	Infrequently prescribed
**Glycerophosphate**	Infrequently prescribed
**Phenylephirine**	Similar to other compounds tested
**Levodopa**	Similar to other compounds tested
**Tetraethylenepentamine**	Component of topical cream – not used systemically
**Carbidopa**	Similar to other compounds tested
**D-glucosamine**	Likelihood of clinically significant interaction low
**Lisdexamfetamine**	Infrequently prescribed
**Ascorbic acid**	Likelihood of clinically significant interaction low
