# Supplementary material for: In silico drug repurposing at the cytoplasmic surface of human aquaporin 1
Source: PLoS One. 2025 Jan 9;20(1):e0314151. doi: 10.1371/journal.pone.0314151 (PMC11717375; doi:10.1371/journal.pone.0314151)
Supplement: Attachment — Submitted filename: 240620 PLOS AQP1 cyto V6.docx [file pone.0314151.s002.docx]

*In silico* drug repurposing at the cytoplasmic surface of human aquaporin 1

Aled R. Lloyd^1^*, Karl Austin-Muttitt^1^, Jonathan G. L. Mullins^1^

^1^ Genome and Structural Bioinformatics Group, Faculty of Medicine, Health and Life Science, Swansea University, Singleton Park, Swansea, Wales, UK, SA2 8PP

# Abstract

Aquaporin 1 is expressed throughout the body and is important for peritoneal dialysis. A computational drug repurposing screen investigating possible inhibitors of water transport has been undertaken.

Docking studies were performed on human AQP1 models assembled using I-TASSER and MODELLER. PLANTS was used to score docking studies of the cytoplasmic side of the protein. Molecular dynamics (MD) studies were prepared using CHARMM-GUI and run using the GROMACS-on-Colab platform. The compound library comprised 1002 approved drug compounds listed in the British National Formulary (BNF).

200 compounds binding in the water pore of AQP1 were identified from docking. MD studies were performed on 30 compounds with the strongest PLANTS binding scores. Of compounds frequently prescribed, dopamine, gabapentin, pregabalin, methyldopa, furosemide and pravastatin remained in putative pore blocking conformations.

Compounds that could inhibit water transport through human AQP1 have been identified in this computational screen. This is potentially relevant to diuretic therapy and peritoneal dialysis.

# Author summary

Aquaporins are involved in water transport across cell membranes and can be found throughout the body. Aquaporin 1 is thought to play an important role in water transport during peritoneal dialysis. Drugs that bind in the water pore of aquaporin could reduce the effectiveness of peritoneal dialysis as an unintended side effect. Using a computer-generated model of human aquaporin 1, we used docking studies to find the best interactions between a library of licensed drug compounds and the protein. A selection of the best interactions were tested by molecular dynamics to assess whether the molecules remained bound to the protein. Potentially significant interactions were identified for dopamine, gabapentin, pregabalin, methyldopa, furosemide and pravastatin. An assessment of the effects of these drug compounds on peritoneal dialysis is warranted.

# Introduction

## Aquaporin 1

Aquaporins are tetrameric proteins. Each protein subunit contains a pore and the whole tetrameric arrangement forms a larger central pore(1). Aquaporin 1 (AQP1) transports water alone through the monomeric pore while single valence cations are transported through the central pore in a process mediated by cGMP(2). Factors governing selectivity for water transport though the pore have been implicated (3–7). Within the nephron, AQP1 is located in the vasa recta, proximal convoluted tubule and the descending limb of the loop of Henle(8). AQP1 is expressed throughout the body and is implicated in water transport during peritoneal dialysis as the ultrasmall pore of the 3 pore model(9). The structure of yeast AQP1 with water molecules in transit was determined by X-ray crystallography in 2013(10).

There is evidence that water transport through AQP1 is inhibited by mercury and tetraethyl ammonium through binding within the intrinsic water pore of the monomer(11). Of currently licensed medication, there is only evidence to support AQP1 inhibition by acetazolamide, bumetanide and furosemide(11–14). Based on this work the loop diuretics act on the intracellular side of the protein, but their precise site and mode of action is unclear with two suggested binding sites. The binding of furosemide was fifteen times more potent than bumetanide(13). Bumetanide derivatives have attracted attention in the field of oncology as GMP mediated cation transport through the central pore of the tetramer is thought to be involved in metastatic spread of some cancers(15). These compounds are believed to act by binding to the AQP1 central pore(16). While the main diuretic action of furosemide is due to inhibition of the sodium/potassium/chloride cotransporter(17), there is also evidence of furosemide interacting with Na^+^/H^+^ exchanger isoform 3 (18) and carbonic anhydrase (19). Additionally, furosemide is known to have anti-inflammatory and anti-asthmatic effects independently of its diuretic properties (20) suggesting that the mode of action of this drug is multifactorial.

It has also been postulated that some of the adverse effects of some antiepileptic medication, such as lamotrigine and topiramate could be due to inhibition of AQP1, although the evidence for this is from extrapolation of oncological studies in mice and studies of other aquaporins(21).

Given that the main clinical applications of AQP1 inhibition relate to water transport, we undertook an *in silico* drug repurposing screen to investigate the binding of existing licensed medication to the cytoplasmic side of the AQP1 water pore. This site was selected as this is an experimentally confirmed route of water transport inhibition(13,14).

Drug repurposing is the identification of new indications or therapeutic approaches for existing licensed medicinal compounds(22,23). It is a cost-efficient option for developing new therapies. Molecular docking is a computational method developed to identify the best energetic fit of an interaction between a small molecule and a large molecule such as a protein(24). The speed at which large numbers of docking simulations can be performed makes docking a cheap and attractive pharmacological research tool, however studies are limited by indiscrimination between active and non-active substances(25). Following the publication of a protocol for high throughput post-docking molecular dynamics studies to improve discrimination between binding compounds and non-binding compounds(25), we have implemented an approach for a drug repurposing molecular docking screen followed by high throughput molecular dynamics studies. This method has been designed to improve accessibility to researchers without advanced computational skills or access to supercomputers by using a notebook based graphical user interface on Google Colaboratory(26).

# Results

## Structural data

### Protein structure modelling

The initial monomeric and tetrameric structures of AQP1 showed very good visual semblance with published crystal structures for human forms of AQP1. The homology model with the water molecules located according to the yeast AQP1 structure 3ZOJ showed good visual agreement with the backbone of the AQP1 system and the relative positions of water molecules in the channel of the yeast structure.

## Docking

Visual inspection of the most favourable conformations for 1002 drug agents from the BNF yielded 198 compounds bound in or near the opening of the cytoplasmic side of the water pore. By restricting these results to only candidates with 3 or more hydrogen bonds which also had calculated binding energies (total energy, energy corrected for molecular weight and energy corrected for rotational loss) greater or equal to that of the known binder furosemide, 45 compounds were identified for further investigation using molecular dynamics approaches.

Figure 1: Flowchart outlining compound selection for molecular dynamic studies from docking study results for all available drug compounds listed in the BNF against the cytoplasmic opening of the water pore of a human AQP1 monomer.

Images from the best docked poses of a selection of compounds can be seen in Figure 2. There is variation in binding sites with gabapentin and dopamine compounds binding deep in the pore interacting by hydrogen bonding with ASN192, while furosemide is found interacting with several residues at the opening of the pore.

In our docking results, the most energetically favourable conformation of furosemide binding to the cytoplasmic opening of the intrinsic pore of the AQP1 monomer is different to predictions previously published(13). The sulphamoyl group of the furosemide molecule binds to a pocket on the cytoplasmic surface of the pore opening with the furan ring protruding into the pore.

We were unable to reproduce the molecular docking findings of Migliati and co-workers with respect to the binding of bumetanide(13). The 10 most energetically favourable conformations for the drug identified by PLANTS were all away from the pore. The most favourable docking conformations of acetazolamide, lamotrigine and topiramate demonstrated interactions along the cytoplasmic chain between residues PRO233 and LYS267.

Figure 2: Panel depicting a selection of the best docked conformations in the water pore of an AQP1 monomer. A – Images of the human AQP1 model with water molecules in transit, B – the best docked conformation of furosemide, C – the best docked conformation of gabapentin, D – the best docked conformation of Dopamine. Predicted hydrogen bonds are represented by green lines.

## Molecular dynamics

A membrane bound AQP1 system was assembled using the membrane builder function of CHARMM-GUI and the model obtained showed agreement with the location of the membrane according to the UniProt database entry P29972for AQP1(30). The 20 nanosecond simulations discriminated between binding and non-binding compounds. As can be seen in the graph of Root Mean Square Deviation (RMSD) over time in Figure 4, all unbound compounds were apparent by 10 nanoseconds.

15 compounds were excluded from the list of 45 either due to similarities to other compounds tested, infrequent clinical use or being endogenous compounds. Table 1 summarises the results of the 30 compounds tested with post-docking MD simulations and the excluded compounds are listed in supplementary data.

Furosemide slowly moved from its best docked position, rotating and interacting with the cytoplasmic chain at the opening of the water pore. A conformational change was observed in the cytoplasmic chain from residue PRO233 to the C-terminus (residue LYS 269) on interacting with the furosemide molecule. This phenomenon is illustrated in Figure 3 and was not observed with any other compounds.

Figure 3: The position of furosemide and the conformational change in a cytoplasmic domain over the duration the MD simulation

Of the compounds with low RMSD values seen in Table 1 only furosemide, levodopa, methyldopa, pravastatin, gabapentin and pregabalin stand out as compounds prescribed frequently. The best docked positions of these compounds are shown in Figure 2.

Table 1: Summary of results of post docking MD studies of drug repurposing screen against the cytoplasmic opening of the water pore of human AQP1

| Compound | Visual inspection | Compound | Visual inspection |
| --- | --- | --- | --- |
| Methyldopa | Bound | **Risendronate** | Not bound |
| Dopamine | Bound | **Propafenone** | Not bound |
| Esmolol | Bound | **Tenoxicam** | Not bound |
| Panobinostat | Bound | **Cytarabine** | Not bound |
| Pregabalin | Bound | **Carbocisteine** | Not bound |
| Gabapentin | Bound | **D-Glucose** | Not bound |
| Procarbazine | Bound | **Safinamide** | Not bound |
| Defersariox | Bound | **Tranexamic acid** | Not bound |
| Glipizide | Bound | **Streptozocin** | Not bound |
| Furosemide | Bound | **Ethambutol** | Not bound |
| Famotidine | Bound | **Metraminol** | Not bound |
| Permetrexed | Bound | **Isosorbide dinitrite** | Not bound |
| Pravastatin | Bound | **Atenolol** | Not bound |
| Chloramphenicol | Bound | **Noradrenaline** | Not bound |
| Fometerol | Bound |  |  |

Methyldopa and dopamine both exhibited very low RMSD values during the simulation. The best docking position of both compounds was deep in the pore and at the site of mercury inhibition. Gabapentin and pregabalin exhibited a similar pattern of binding.

Root mean square deviation (Angstroms)

| Time (nanoseconds) | Atenolol | Furosemide | Gabapentin | Glipizide | Isosorbide dinitrite | Metraminol | Pravastatin | Pregablin | Procarbazine | Risendronate | Tranexamic acid | Noradrenaline | Dopamine | Methyldopa | Carbocisteine | Famotidine |
| --- | --- | --- | --- | --- | --- | --- | --- | --- | --- | --- | --- | --- | --- | --- | --- | --- |
| 0 | 1e-06 | 1.4e-05 | 0 | 1e-06 | 1e-06 | 0 | 1e-06 | 1e-06 | 1e-06 | 1e-06 | 1e-06 | 1e-06 | 1e-06 | 1e-06 | 8e-06 | 6e-06 |
| 1 | 4.09085 | 1.91973 | 1.07373 | 2.10224 | 4.39574 | 7.35992 | 3.43902 | 1.99673 | 1.68248 | 8.6068 | 1.51911 | 5.46825 | 1.74125 | 1.25686 | 0.69216 | 2.77698 |
| 2 | 10.2484 | 2.55458 | 1.02129 | 2.29364 | 7.7381 | 8.2954 | 2.97141 | 1.91576 | 1.0001 | 6.05764 | 5.21678 | 6.11632 | 1.50698 | 1.14878 | 0.94974 | 2.94695 |
| 3 | 10.4785 | 2.31052 | 1.61117 | 2.3661 | 16.0694 | 9.23181 | 3.10645 | 2.35698 | 1.00265 | 6.84859 | 6.35763 | 5.12234 | 1.62928 | 0.855765 | 7.31774 | 3.3338 |
| 4 | 8.37697 | 2.0455 | 1.4988 | 3.49467 |  | 7.91723 | 4.91479 | 1.95295 | 2.30701 | 7.5456 | 7.36308 | 5.73234 | 1.99147 | 1.84841 | 7.32565 | 3.5356 |
| 5 | 8.48151 | 2.27745 | 2.11227 | 2.18444 |  | 9.58981 | 4.26944 | 2.32181 | 1.01106 | 7.44226 | 9.64613 | 9.52224 | 0.978292 | 1.22052 | 7.46141 | 3.66082 |
| 6 | 9.35203 | 4.6722 | 2.06454 | 3.16016 |  | 11.2669 | 4.53179 | 2.26922 | 1.94846 | 6.88279 | 7.88218 | 8.82914 | 1.36449 | 1.02435 | 10.3865 | 3.73599 |
| 7 | 15.5174 | 4.10459 | 1.82952 | 2.90328 |  |  | 4.07272 | 1.87595 | 1.51763 | 6.97284 | 9.11873 | 9.3593 | 0.869143 | 1.00845 | 11.606 | 3.37205 |
| 8 |  | 4.36652 | 1.82555 | 3.18063 |  |  | 4.49786 | 1.75764 | 2.10387 | 7.95668 | 9.55987 | 9.94863 | 1.46036 | 1.3683 | 8.8764 | 4.0957 |
| 9 |  | 4.8597 | 2.18478 | 4.09363 |  |  | 4.53958 | 2.28671 | 2.0712 | 7.77364 | 10.2102 | 9.73062 | 1.24676 | 1.12582 | 14.0913 | 2.27574 |
| 10 |  | 4.04742 | 1.84773 | 3.28466 |  |  | 5.70945 | 2.57351 | 1.3023 | 7.5954 | 12.2411 | 6.28873 | 0.968441 | 1.26901 |  | 2.61928 |
| 11 |  | 3.8073 | 2.01222 | 3.03585 |  |  | 4.98959 | 2.56372 | 1.8095 | 7.3729 |  | 6.00618 | 0.675073 | 0.774391 |  | 3.26581 |
| 12 |  | 4.11741 | 2.09613 | 3.53608 |  |  | 4.905 | 2.3992 | 2.20307 | 7.15059 |  | 6.03223 | 1.20669 | 1.0667 |  | 3.581 |
| 13 |  | 4.07847 | 2.44329 | 3.27219 |  |  | 5.00759 | 2.50578 | 2.27616 | 8.82471 |  | 5.76966 | 1.18294 | 1.5389 |  | 3.11196 |
| 14 |  | 5.79522 | 2.24864 | 3.05904 |  |  | 5.63159 | 1.89089 | 1.38655 | 9.8106 |  | 6.19099 | 1.17601 | 1.35429 |  | 2.63949 |
| 15 |  | 5.44894 | 1.88445 | 3.10113 |  |  | 4.61467 | 1.84745 | 1.88526 | 8.06726 |  | 6.79367 | 0.699755 | 1.45489 |  | 3.98146 |

Figure 4: RMSD over time for a selection of compounds included in the MD simulations. The compounds assessed as not being bound are indicated with dotted lines, while thick lines indicate bound complexes commonly prescribed.

The best docking conformation for pravastatin was at the opening of the water pore in a position likely to occlude the flow of water. As the MD simulation progressed, the molecule rotated but otherwise remained over the pore opening.

# Discussion

We hypothesised that potential new therapeutic opportunities could be identified by performing a drug repurposing screen at the cytoplasmic opening of the AQP1 water pore. The potential clinical applications of AQP inhibition include treating conditions such as volume overload related to cardiac or renal dysfunction, the treatment of glaucoma or macular oedema and even potentially cerebral oedema due to stroke(11). As a result there is an emerging effort to identify inhibitors at scale (49). In contrast to other studies, this work has focussed on existing licensed medication with the associated possibility that adverse effects may also be explained by identifying an interaction. There are circumstances where AQP1 inhibition would be undesirable such as peritoneal dialysis, where AQP1 is thought to play a prominent role(9).

The evidence for AQP1 inhibition by existing licensed medicinal compounds is relatively sparse. Furosemide and bumetanide inhibit water transport through AQP1 by binding to the cytoplasmic side of the protein. Direct binding to the pore and a regulatory action through binding to a cytoplasmic chain have both been proposed as explanations for this effect(12,13). In this study furosemide moved away from a putative docked position at the opening of the pore eventually settling in a position where a conformational change in the cytoplasmic chain was induced. Based on this simulation, furosemide appears to have several relatively stable binding positions on the cytoplasmic surface of human AQP1 and interacts with the cytoplasmic chain in a way not observed in any of the other compounds that underwent MD simulations.

The choice of search radius in this study was in part influenced by the suggestion that bumetanide could occlude the cytoplasmic opening of the water pore(13), while ensuring that the site of mercury and tetraethylammonium inhibition was also studied(14). The fact that we were not able to generate an energetically favourable pose for bumetanide within the pore or the cytoplasmic chain of AQP1 reflects its preference in our simulation for hydrophobic interactions. The difference in binding is in keeping with experimental findings that the binding of bumetanide to AQP1 is 15 times weaker than the binding of furosemide(13). Bumetanide derivatives have attracted attention in oncology where tubule formation and the migration of cancer cells can be reduced(15,16). *In silico* work related to these compounds suggests they bind at the opening of the central ion pore and that their effects are due to the inhibition of cation transport rather than water(16). The variation in optimal docked poses of the compounds in this repurposing screen is interesting. The intrinsic pore of the AQP1 monomer can accommodate six membered rings, and unsurprisingly for a protein involved in the rapid transfer of a chain of water molecules, provides ample hydrogen bonding opportunities. This accounts for the stable pose of gabapentin in the simulations described here. In general, larger molecules forming multiple hydrogen bonds at the cytoplasmic opening of the water pore were less stable in MD simulations where rotational and entropic factors feature more prominently.

Based on our work it is not possible to characterise the exact nature of water transport inhibition by loop diuretics on AQP1, but possible explanations include a regulatory effect by binding to a cytoplasmic region or direct inhibition via a dynamic process involving multiple sites of interaction.

There is an open question as to whether acetazolamide, lamotrigine, and topiramate affect water transport through AQP1(14,21). If these compounds do affect water transport through AQP1, based on our work, their site of action would be away from the cytoplasmic side of the water pore. The best docked conformations of acetazolamide and lamotrigine identified in this study were in the cytoplasmic region of AQP1. It is interesting that dopamine-based compounds and gabapentinoids are believed to mainly act within the nervous system and it is possible that inhibiting CNS aquaporins may contribute to the observed effects of these drugs.

Interactions between AQP1 and any of methyldopa, levodopa gabapentin, pregabalin or pravastatin are a novel finding. None of these compounds have side effects associated with volume depletion(50), or have ever been investigated for their diuretic properties or for reductions in peritoneal dialysis transport.

AQP1 interacted with a range of different types of compounds in this study, mostly some smaller compounds capable of forming multiple hydrogen bonds, suggesting an element of promiscuity for different ligands. This idea is something that has been studied in kinases(51) and in the case of the cytoplasmic opening of the AQP1 pore is likely the result of an abundance of hydrogen bond donors and acceptors on the protein, and a generous pore diameter near the opening that can accommodate six membered rings.

The user-friendly GROMACS-on-Colab system we have developed for the integration of docked ligands using CHARMM-GUI followed by Google Colab based GROMACS simulation performed reliably with a wide range of ligands. However, the computational nature of this work means that *in vivo* factors such as pharmacokinetics and pharmacodynamics are not considered. While docking studies give the best energetic fit of a ligand with a protein there is no guarantee that the ligand would occupy that position in life. We have only considered the ligand conformation with the best PLANTS score; it is possible that we have not included plausible interactions where a compound might have several favourable binding positions.

Corroboration of our findings with experimental work in relation to the cytoplasmic opening of the human AQP1 water pore and investigation of drug binding to the cytoplasmic chains of the protein, particularly the region associated with cGMP binding, are the next steps we recommend following on from this work.

# Materials and Methods

## Structural modelling

As portions of the cytoplasmic chains were incomplete in crystal structures and in order to create a model based solely on human sequences, threading modelling using the I-TASSER server and suite(27–29) was performed using the canonical FASTA sequence for AQP1 on the UniProt database(entry P29972)(30). Structural visualisation and analysis were performed using UCSF Chimera software versions 1.16 and X1.2.5(31). Oligomerisation of the monomeric structure obtained from I-TASSER was performed by homology modelling using MODELLER(32).

### Modelling of water in an aquaporin channel

We generated a model of human AQP1 with water molecules within the water pore using the position of water molecules from yeast aquaporin (PDB:3ZOJ) (33) to ensure that protein side chain orientation was compatible with water transport for the docking studies. Fifteen water molecules in the 3ZOJ(10) structure were identified for the modelling of pore water and quaternary structure. The ParMATT structural alignment algorithm was used to align 3ZOJ and our AQP1 model(33). Protein residues within 4 Angstroms of these 15 water molecules were identified from the 3ZOJ structure and maintained. MODELLER (32) was used to generate a structure based on the I-TASSER structure of AQP1 and the restricted model of 3ZOJ.

### Membrane site identification

The membrane builder function of CHARM-GUI was used for building a lipid bilayer around the assembled tetramer(34–38).

## Docking studies

### Library of Compounds

3D structures of all 1512 medications listed in the British National Formulary were sought from the NCBI PubChem databases(39). Once duplicate records and substances for which there was no structure on the PubChem database were excluded, 1002 medicinal compounds were left in the screening library. Where a compound was listed as a mixture; all components of the mixture were sought from the database. The most common reason a structure could not be found was due to the agent’s large molecular size with medicines such as vaccines, monoclonal antibodies and haematological products featuring prominently in this group. The list of compounds included and excluded can be found in appendix 1.

### Active site identification

The cytoplasmic opening of the intrinsic pore of the AQP1 monomer was identified as the site for investigation as drug binding at this location would impede water transport. Evidence that loop diuretics act on the cytoplasmic surface of AQP1 informed this decision(12,13). A wide radius of study (20Å) was selected as no data on precise binding to the cytoplasmic opening is available. The radius of study incorporated the cytoplasmic half of the water pore, the site of mercury inhibition, most of the cytoplasmic chains and membrane regions of the protein.

Figure 5: Human AQP1 monomer with residues within docking search area highlighted in red. Approximate position of membrane shown by dotted lines.

### Drug binding analysis

After converting file formats using Open Babel(40), PLANTS software was used to establish the most energetically favourable binding positions of each compound(41,42). The binding regions of these structures were classified by visual inspection using UCSF Chimera(31) followed by interrogation of the PLANTS scores for binding energy(42) to establish which compounds were most likely to exhibit a pore blocking interaction with the protein(31). These processes filtered the docking outcomes to a smaller number for further testing using MD.

## MD studies

We have developed a bespoke cloud based system for preparing MD input files using CHARMM-GUI(34,35,37,38,43) and running MD simulations using GROMACS(44,45) entirely on graphics processing units in Google Colaboratory(26).

To prepare the MD system, a membrane bound protein system was assembled using Membrane Builder function of CHARMM-GUI (34–36,46) and docked ligands prepared using the Ligand Reader and Modeler feature of CHARMM-GUI(47). The topology and coordinate files were merged and overlapping solvent molecules deleted. We used the TIP3 model for water and POPC to represent the phospholipid bilayer. The energy minimisation and equilibration steps of a GROMACS simulation were then run on a GPU as part of the same system preparation notebook.

15 nanosecond production MD simulations were undertaken to determine which docked ligand poses were the most stable. The CHARMM36 forcefield was used with a pressure of 1 bar and a temperature of 303.15 K. An early stop mechanism was incorporated within the simulation for the point where the root mean square deviation of the ligand exceeds 10Å. Analysis of the MD results was carried out by visual inspection using VMD software(48) and graph analysis of RMSD over time. The RMSD is a median measurement of the displacement between protein and ligand relative to the starting position of the MD simulation. In these simulations the starting position for the ligand is the best conformation in the docking study.

# Conclusion

Our computational analysis has identified compounds that could interfere with water transport through interaction with human AQP1, including furosemide, methyldopa, levodopa, gabapentin, pregabalin or pravastatin. This is potentially of great relevance to diuretic therapy and peritoneal dialysis and the work will help inform the *in vitro* and *in vivo* studies necessary to ultimately confirm these protein-ligand interactions of potentially substantial clinical significance.

# References

1.	Ozu M, Galizia L, Acuña C, Amodeo G. Aquaporins: More Than Functional Monomers in a Tetrameric Arrangement. Cells. 2018 Nov 11;7(11):209.

2.	Boassa D, Yool AJ. A fascinating tail: cGMP activation of aquaporin-1 ion channels. Trends Pharmacol Sci. 2002 Dec;23(12):558–62.

3.	Chakrabarti N, Tajkhorshid E, Roux B, Pomès R. Molecular Basis of Proton Blockage in Aquaporins. Structure. 2004 Mar;12(1):65–74.

4.	Li H, Chen H, Steinbronn C, Wu B, Beitz E, Zeuthen T, et al. Enhancement of proton conductance by mutations of the selectivity filter of aquaporin-1. J Mol Biol. 2011 Apr 8;407(4):607–20.

5.	Ilan B, Tajkhorshid E, Schulten K, Voth GA. The mechanism of proton exclusion in aquaporin channels. Proteins. 2004 May 1;55(2):223–8.

6.	Beitz E, Pavlovic-Djuranovic S, Yasui M, Agre P, Schultz JE. Molecular dissection of water and glycerol permeability of the aquaglyceroporin from Plasmodium falciparum by mutational analysis. Proc Natl Acad Sci U S A. 2004 Feb 3;101(5):1153–8.

7.	Beitz E, Wu B, Holm LM, Schultz JE, Zeuthen T. Point mutations in the aromatic/arginine region in aquaporin 1 allow passage of urea, glycerol, ammonia, and protons. Proc Natl Acad Sci U S A. 2006 Jan 10;103(2):269–74.

8.	He J, Yang B. Aquaporins in Renal Diseases. IJMS. 2019 Jan 16;20(2):366.

9.	Piccapane F, Gerbino A, Carmosino M, Milano S, Arduini A, Debellis L, et al. Aquaporin-1 Facilitates Transmesothelial Water Permeability: In Vitro and Ex Vivo Evidence and Possible Implications in Peritoneal Dialysis. IJMS. 2021 Nov 21;22(22):12535.

10.	Kosinska Eriksson U, Fischer G, Friemann R, Enkavi G, Tajkhorshid E, Neutze R. Subangstrom Resolution X-Ray Structure Details Aquaporin-Water Interactions. Science. 2013 Jun 14;340(6138):1346–9.

11.	Salman MM, Kitchen P, Yool AJ, Bill RM. Recent breakthroughs and future directions in drugging aquaporins. Trends in Pharmacological Sciences. 2022 Jan;43(1):30–42.

12.	Ozu M, Dorr RA, Teresa Politi M, Parisi M, Toriano R. Water flux through human aquaporin 1: inhibition by intracellular furosemide and maximal response with high osmotic gradients. Eur Biophys J. 2011 Jun;40(6):737–46.

13.	Migliati E, Meurice N, DuBois P, Fang JS, Somasekharan S, Beckett E, et al. Inhibition of aquaporin-1 and aquaporin-4 water permeability by a derivative of the loop diuretic bumetanide acting at an internal pore-occluding binding site. Mol Pharmacol. 2009 Jul;76(1):105–12.

14.	Yang B, Kim JK, Verkman AS. Comparative efficacy of HgCl _2_ with candidate aquaporin-1 inhibitors DMSO, gold, TEA ^+^ and acetazolamide. FEBS Letters. 2006 Dec 11;580(28–29):6679–84.

15.	Tomita Y, Palethorpe HM, Smith E, Nakhjavani M, Townsend AR, Price TJ, et al. Bumetanide-Derived Aquaporin 1 Inhibitors, AqB013 and AqB050 Inhibit Tube Formation of Endothelial Cells through Induction of Apoptosis and Impaired Migration In Vitro. Int J Mol Sci. 2019 Apr 12;20(8):1818.

16.	Kourghi M, Pei JV, De Ieso ML, Flynn G, Yool AJ. Bumetanide Derivatives AqB007 and AqB011 Selectively Block the Aquaporin-1 Ion Channel Conductance and Slow Cancer Cell Migration. Mol Pharmacol. 2016 Jan;89(1):133–40.

17.	Moseng MA, Su CC, Rios K, Cui M, Lyu M, Glaza P, et al. Inhibition mechanism of NKCC1 involves the carboxyl terminus and long-range conformational coupling. Sci Adv. 2022 Oct 28;8(43):eabq0952.

18.	De Bruijn PIA, Larsen CK, Frische S, Himmerkus N, Praetorius HA, Bleich M, et al. Furosemide-induced urinary acidification is caused by pronounced H ^+^ secretion in the thick ascending limb. American Journal of Physiology-Renal Physiology. 2015 Jul 15;309(2):F146–53.

19.	Huang X, Dorhout Mees E, Vos P, Hamza S, Braam B. Everything we always wanted to know about furosemide but were afraid to ask. Am J Physiol Renal Physiol. 2016 May 1;310(10):F958-971.

20.	Prandota J. Furosemide: progress in understanding its diuretic, anti-inflammatory, and bronchodilating mechanism of action, and use in the treatment of respiratory tract diseases. Am J Ther. 2002 Aug;9(4):317–28.

21.	Shank RP, Maryanoff BE. Molecular pharmacodynamics, clinical therapeutics, and pharmacokinetics of topiramate. CNS Neurosci Ther. 2008;14(2):120–42.

22.	Roessler HI, Knoers NVAM, van Haelst MM, van Haaften G. Drug Repurposing for Rare Diseases. Trends Pharmacol Sci. 2021 Apr;42(4):255–67.

23.	Mullins JGL. Drug repurposing in silico screening platforms. Biochem Soc Trans. 2022 Apr 29;50(2):747–58.

24.	Pinzi L, Rastelli G. Molecular Docking: Shifting Paradigms in Drug Discovery. Int J Mol Sci. 2019 Sep 4;20(18):4331.

25.	Guterres H, Im W. Improving Protein-Ligand Docking Results with High-Throughput Molecular Dynamics Simulations. J Chem Inf Model. 2020 Apr 27;60(4):2189–98.

26.	Bisong E. Building Machine Learning and Deep Learning Models on Google Cloud Platform: A Comprehensive Guide for Beginners [Internet]. Berkeley, CA: Apress; 2019 [cited 2023 Jul 26]. Available from: http://link.springer.com/10.1007/978-1-4842-4470-8

27.	Yang J, Zhang Y. I-TASSER server: new development for protein structure and function predictions. Nucleic Acids Res. 2015 Jul 1;43(W1):W174-181.

28.	Yang J, Yan R, Roy A, Xu D, Poisson J, Zhang Y. The I-TASSER Suite: protein structure and function prediction. Nat Methods. 2015 Jan;12(1):7–8.

29.	Zheng W, Zhang C, Li Y, Pearce R, Bell EW, Zhang Y. Folding non-homologous proteins by coupling deep-learning contact maps with I-TASSER assembly simulations. Cell Rep Methods. 2021 Jul 26;1(3):100014.

30.	The UniProt Consortium, Bateman A, Martin MJ, Orchard S, Magrane M, Agivetova R, et al. UniProt: the universal protein knowledgebase in 2021. Nucleic Acids Research. 2021 Jan 8;49(D1):D480–9.

31.	Pettersen EF, Goddard TD, Huang CC, Couch GS, Greenblatt DM, Meng EC, et al. UCSF Chimera--a visualization system for exploratory research and analysis. J Comput Chem. 2004 Oct;25(13):1605–12.

32.	Eswar N, Webb B, Marti-Renom MA, Madhusudhan MS, Eramian D, Shen MY, et al. Comparative protein structure modeling using Modeller. Curr Protoc Bioinformatics. 2006 Oct;Chapter 5:Unit-5.6.

33.	Shegay MV, Suplatov DA, Popova NN, Švedas VK, Voevodin VV. parMATT: parallel multiple alignment of protein 3D-structures with translations and twists for distributed-memory systems. Bioinformatics. 2019 Nov 1;35(21):4456–8.

34.	Wu EL, Cheng X, Jo S, Rui H, Song KC, Dávila-Contreras EM, et al. CHARMM-GUI Membrane Builder toward realistic biological membrane simulations. J Comput Chem. 2014 Oct 15;35(27):1997–2004.

35.	Jo S, Lim JB, Klauda JB, Im W. CHARMM-GUI Membrane Builder for mixed bilayers and its application to yeast membranes. Biophys J. 2009 Jul 8;97(1):50–8.

36.	Jo S, Kim T, Im W. Automated builder and database of protein/membrane complexes for molecular dynamics simulations. PLoS One. 2007 Sep 12;2(9):e880.

37.	Lee J, Patel DS, Ståhle J, Park SJ, Kern NR, Kim S, et al. CHARMM-GUI Membrane Builder for Complex Biological Membrane Simulations with Glycolipids and Lipoglycans. J Chem Theory Comput. 2019 Jan 8;15(1):775–86.

38.	Jo S, Kim T, Iyer VG, Im W. CHARMM-GUI: a web-based graphical user interface for CHARMM. J Comput Chem. 2008 Aug;29(11):1859–65.

39.	Kim S, Chen J, Cheng T, Gindulyte A, He J, He S, et al. PubChem in 2021: new data content and improved web interfaces. Nucleic Acids Research. 2021 Jan 8;49(D1):D1388–95.

40.	O’Boyle NM, Banck M, James CA, Morley C, Vandermeersch T, Hutchison GR. Open Babel: An open chemical toolbox. J Cheminform. 2011 Dec;3(1):33.

41.	Dorigo M, editor. Ant colony optimization and swarm intelligence: 5th international workshop, ANTS 2006, Brussels, Belgium, September 4-7, 2006: proceedings. Berlin ; New York: Springer; 2006. 526 p. (Lecture notes in computer science).

42.	Korb O, Stützle T, Exner TE. Empirical scoring functions for advanced protein-ligand docking with PLANTS. J Chem Inf Model. 2009 Jan;49(1):84–96.

43.	Lee J, Cheng X, Swails JM, Yeom MS, Eastman PK, Lemkul JA, et al. CHARMM-GUI Input Generator for NAMD, GROMACS, AMBER, OpenMM, and CHARMM/OpenMM Simulations Using the CHARMM36 Additive Force Field. J Chem Theory Comput. 2016 Jan 12;12(1):405–13.

44.	Abraham MJ, Murtola T, Schulz R, Páll S, Smith JC, Hess B, et al. GROMACS: High performance molecular simulations through multi-level parallelism from laptops to supercomputers. SoftwareX. 2015 Sep;1–2:19–25.

45.	Pronk S, Páll S, Schulz R, Larsson P, Bjelkmar P, Apostolov R, et al. GROMACS 4.5: a high-throughput and highly parallel open source molecular simulation toolkit. Bioinformatics. 2013 Apr 1;29(7):845–54.

46.	Lee J, Patel DS, Ståhle J, Park SJ, Kern NR, Kim S, et al. CHARMM-GUI *Membrane Builder* for Complex Biological Membrane Simulations with Glycolipids and Lipoglycans. J Chem Theory Comput. 2019 Jan 8;15(1):775–86.

47.	Kim S, Lee J, Jo S, Brooks CL, Lee HS, Im W. CHARMM-GUI ligand reader and modeler for CHARMM force field generation of small molecules: CHARMM-GUI Ligand Reader and Modeler for CHARMM Force Field Generation of Small Molecules. J Comput Chem. 2017 Jun 5;38(21):1879–86.

48.	Humphrey W, Dalke A, Schulten K. VMD: visual molecular dynamics. J Mol Graph. 1996 Feb;14(1):33–8, 27–8.

49.	Patil RV, Xu S, van Hoek AN, Rusinko A, Feng Z, May J, et al. Rapid Identification of Novel Inhibitors of the Human Aquaporin-1 Water Channel. Chem Biol Drug Des. 2016 May;87(5):794–805.

50.	BNF 86: September 2023-March 2024. London: Pharmaceutical Press; 2023.

51.	Hanson SM, Georghiou G, Thakur MK, Miller WT, Rest JS, Chodera JD, et al. What Makes a Kinase Promiscuous for Inhibitors? Cell Chemical Biology. 2019 Mar;26(3):390-399.e5.

# Supplementary data

Table 2: Compounds excluded from MD study at mid-study review and the reason for exclusion.

| Compound | Reason for exclusion |
| --- | --- |
| Acebutolol | Infrequently prescribed |
| Dobutamine | Similar to other compounds tested |
| Triethylenetetramine | Infrequently prescribed |
| Azelaic acid | Infrequently prescribed |
| Glucuronic acid | Infrequently prescribed |
| Glycerophosphate | Infrequently prescribed |
| Phenylephirine | Similar to other compounds tested |
| Levodopa | Similar to other compounds tested |
| Tetraethylenepentamine | Component of topical cream – not used systemically |
| Carbidopa | Similar to other compounds tested |
| D-glucosamine | Likelihood of clinically significant interaction low |
| Lisdexamfetamine | Infrequently prescribed |
| Ascorbic acid | Likelihood of clinically significant interaction low |
