# Supplementary material for: In silico drug repurposing at the cytoplasmic surface of human aquaporin 1
Source: PLoS One. 2025 Jan 9;20(1):e0314151. doi: 10.1371/journal.pone.0314151 (PMC11717375; doi:10.1371/journal.pone.0314151)
Supplement: Attachment — Submitted filename: 240918_Reviewer_Response.docx [file pone.0314151.s003.docx]

# Editor Comments

We thank the editor for their comments and clarification of requirements. In response:

1. The article format has been altered to comply with the journal specifications

2. The code used for MD simulations is freely available through github at <https://github.com/bioinfkaustin/gromacs-on-colab>.

3. References to figure 1 and figure 5 can now be found in the text.

4. The supplementary data has been moved to a separate file as requested

# Reviewer 1

We thank Reviewer 1, Dr Rout, for his comments. In response:

1. The abstract has been entirely rewritten.
2. The requested additions have been made to the introduction
3. This information can be found in the methods and results section of the revised report.
4. We acknowledge that through reporting only 15 nanoseconds of data, the molecular dynamics (MD) elements of this study include relatively short simulations. This important aspect of study design was not a choice taken lightly. MD studies are a valuable addition to the assessment of docking results and have been validated for this purpose^1^. A validation study using the DUD-E dataset identified a root mean square deviation (RMSD) threshold of 5.5Å was robust in discerning true active poses in MD simulations of 10, 50 and 100 nanosecond durations. In this study the difference in sensitivity measured by the area under a ROC curve (AUC) between each simulation length was relatively small. The calculated AUC values for experiments using the DUD-E dataset were 0.806, 0.840 and 0.836 for simulations of 10, 50 and 100 nanoseconds respectively(25).
   The current study is the first performed using a study protocol for high-throughput docking-MD screening that aims to make such structural bioinformatics studies achievable and accessible to new researchers to the field and researchers in training. This study is not alone in selecting a short duration of MD simulation to evaluate docking results with over 50 examples of studies using 10 nanosecond simulations identified on the NCBI PubMed database at the time of writing^2–53^.
5. Despite a thorough literature review, we have been unable to identify studies validating the use of root mean square fluctuation (RMSF) and RF in short post-docking MD studies. Without this validation it is unclear whether these additional measurements would meaningfully enhance the study conclusions. As outlined above increasing the complexity of data analysis is contrary to the premise of the protocol under development. In response to the points made, more detail has been included regarding selected observed hydrogen bond patterns before and after the MD simulations which can be found in a dedicated section within the results.
6. A dedicated section has been included with information on the validation of the model. This includes confidence scores obtained from I-TASSER and RMSD values against reference structures.
7. As explained in our response to point 4 (above), with the validation provided and strong support across a raft of literature, we sincerely believe that creditable conclusions can be made from 10 nanosecond simulations in this validated setting^1^, see point 4 above. We accept that wherever possible, the results of any computational study, but especially those investigating ligand binding, should be validated by laboratory experiments or clinical data due to the inherent limitations of the methods. Both the original and rewritten conclusions make this point plainly.
8. The discussion has been extended by the inclusion of a section discussing the rationale for the methods used.

# Reviewer 2

We thank Reviewer 2 for their comments. In response:

1. A robust defence of the methods employed within this study and their rationale can be found above.
2. The abstract and conclusion sections have been entirely rewritten. Redrafting these sections has taken place without knowledge of the specific concerns of the reviewers and we hope that the new versions are satisfactory. More widely, we have also extended the introduction, including additional references to appropriate studies.
3. We assume that the “recent relevant paper” referred to here is the same investigation of a catfish aquaporin recommended by Reviewer 1^54^. We have referred to this article in the text.

Suggestions within the document

4. The abstract has been completely rewritten given uncertainty about which elements of the abstract required improvement.
5. We have extended the introduction, including additional references to appropriate studies.
6. We again assume that the recent publication referenced here is the mechanistic study of water transport through catfish aquaporin by Behera and colleagues 2022^54^. This study investigating the mechanism of water transport through AQP1 is fundamentally different in its aims and design to the assessment of stability of docked ligand conformations in a screening study. As explained in the response to reviewer 1 the differences between 10 and 100 nanosecond MD simulations for discerning true binding from docking study results are modest^1^.
7. The conclusion has been rewritten as mentioned in point 3.

# References

1.	Guterres, H. & Im, W. Improving Protein-Ligand Docking Results with High-Throughput Molecular Dynamics Simulations. *J. Chem. Inf. Model.* **60**, 2189–2198 (2020).

2.	Agarwal, S. *et al.* An integrated computational approach of molecular dynamics simulations, receptor binding studies and pharmacophore mapping analysis in search of potent inhibitors against tuberculosis. *J Mol Graph Model* **83**, 17–32 (2018).

3.	Ali, M. A., Nath, A., Jannat, M. & Islam, M. M. Direct Synthesis of Diamides from Dicarboxylic Acids with Amines Using Nb2O5 as a Lewis Acid Catalyst and Molecular Docking Studies as Anticancer Agents. *ACS Omega* **6**, 25002–25009 (2021).

4.	Aliko, V. *et al.* ‘From shadows to shores’-quantitative analysis of CuO nanoparticle-induced apoptosis and DNA damage in fish erythrocytes: A multimodal approach combining experimental, image-based quantification, docking and molecular dynamics. *Sci Total Environ* **906**, 167698 (2024).

5.	Aloui, M. *et al.* QSAR modelling, molecular docking, molecular dynamic and ADMET prediction of pyrrolopyrimidine derivatives as novel Bruton’s tyrosine kinase (BTK) inhibitors. *Saudi Pharm J* **32**, 101911 (2024).

6.	Amini, R., Moradi, S., Najafi, R., Mazdeh, M. & Taherkhani, A. BACE1 Inhibition Utilizing Organic Compounds Holds Promise as a Potential Treatment for Alzheimer’s and Parkinson’s Diseases. *Oxid Med Cell Longev* **2024**, 6654606 (2024).

7.	Askari, F. S. *et al.* Digging for the discovery of SARS-CoV-2 nsp12 inhibitors: a pharmacophore-based and molecular dynamics simulation study. *Future Virol* (2022) doi:10.2217/fvl-2022-0054.

8.	Azam, M. A. & Jupudi, S. Insight into the structural requirements of thiophene-3-carbonitriles-based MurF inhibitors by 3D-QSAR, molecular docking and molecular dynamics study. *J Recept Signal Transduct Res* **37**, 522–534 (2017).

9.	Azam, M. A. & Thathan, J. Pharmacophore generation, atom-based 3D-QSAR and molecular dynamics simulation analyses of pyridine-3-carboxamide-6-yl-urea analogues as potential gyrase B inhibitors. *SAR QSAR Environ Res* **28**, 275–296 (2017).

10.	Bandaru, S. *et al.* Molecular dynamic simulations reveal suboptimal binding of salbutamol in T164I variant of β2 adrenergic receptor. *PLoS One* **12**, e0186666 (2017).

11.	Chaudhuri, A., Bera, A. K., Sarkar, I. & Chakraborty, S. Insights from Analysis of Binding Sites of Human Meprins: Screening of Inhibitors by Molecular Dynamics Simulation Study. *Comb Chem High Throughput Screen* **19**, 246–258 (2016).

12.	Chauhan, D. *et al.* Design, synthesis, biological evaluation, and molecular modeling studies of rhodanine derivatives as pancreatic lipase inhibitors. *Arch Pharm (Weinheim)* **352**, e1900029 (2019).

13.	Chen, P.-Y. & Han, L.-T. Study on the molecular mechanism of anti-liver cancer effect of Evodiae fructus by network pharmacology and QSAR model. *Front Chem* **10**, 1060500 (2022).

14.	da Silva, G. D. *et al.* In vitro and in silico studies of the larvicidal and anticholinesterase activities of berberine and piperine alkaloids on Rhipicephalus microplus. *Ticks Tick Borne Dis* **12**, 101643 (2021).

15.	Das, B. K., Pv, P. & Chakraborty, D. Computational insights into factor affecting the potency of diaryl sulfone analogs as Escherichia coli dihydropteroate synthase inhibitors. *Comput Biol Chem* **78**, 37–52 (2019).

16.	Dhameliya, T. M., Nagar, P. R. & Gajjar, N. D. Systematic virtual screening in search of SARS CoV-2 inhibitors against spike glycoprotein: pharmacophore screening, molecular docking, ADMET analysis and MD simulations. *Mol Divers* **26**, 2775–2792 (2022).

17.	Gahtori, J., Pant, S. & Srivastava, H. K. Modeling antimalarial and antihuman African trypanosomiasis compounds: a ligand- and structure-based approaches. *Mol Divers* **24**, 1107–1124 (2020).

18.	Guerrero-Perilla, C., Bernal, F. A. & Coy-Barrera, E. Insights into the interaction and binding mode of a set of antifungal azoles as inhibitors of potential fungal enzyme-based targets. *Mol Divers* **22**, 929–942 (2018).

19.	Halder, S. K. & Elma, F. In silico identification of novel chemical compounds with antituberculosis activity for the inhibition of InhA and EthR proteins from Mycobacterium tuberculosis. *J Clin Tuberc Other Mycobact Dis* **24**, 100246 (2021).

20.	Huang, X., Dorhout Mees, E., Vos, P., Hamza, S. & Braam, B. Everything we always wanted to know about furosemide but were afraid to ask. *Am J Physiol Renal Physiol* **310**, F958-971 (2016).

21.	Jamal, S., Grover, A. & Grover, S. Machine Learning From Molecular Dynamics Trajectories to Predict Caspase-8 Inhibitors Against Alzheimer’s Disease. *Front Pharmacol* **10**, 780 (2019).

22.	Johari, S., Sharma, A., Sinha, S. & Das, A. Integrating pharmacophore mapping, virtual screening, density functional theory, molecular simulation towards the discovery of novel apolipoprotein (apoE ε4) inhibitors. *Comput Biol Chem* **79**, 83–90 (2019).

23.	Kamel, E. M. *et al.* Molecular modeling and DFT studies on the antioxidant activity of Centaurea scoparia flavonoids and molecular dynamics simulation of their interaction with β-lactoglobulin. *RSC Adv* **13**, 12361–12374 (2023).

24.	Kamel, E. M. *et al.* Xanthine Oxidase Inhibitory Activity of Euphorbia peplus L. Phenolics. *Comb Chem High Throughput Screen* **25**, 1336–1344 (2022).

25.	Kausar, M. A. *et al.* Identifying the alpha-glucosidase inhibitory potential of dietary phytochemicals against diabetes mellitus type 2 via molecular interactions and dynamics simulation. *Cell Mol Biol (Noisy-le-grand)* **67**, 16–26 (2022).

26.	Khan, A. A., Baildya, N., Dutta, T. & Ghosh, N. N. Inhibitory efficiency of potential drugs against SARS-CoV-2 by blocking human angiotensin converting enzyme-2: Virtual screening and molecular dynamics study. *Microb Pathog* **152**, 104762 (2021).

27.	Khan, M. K. A., Akhtar, S. & Arif, J. M. Development of In Silico Protocols to Predict Structural Insights into the Metabolic Activation Pathways of Xenobiotics. *Interdiscip Sci* **10**, 329–345 (2018).

28.	Khan, M. F. *et al.* Dibenzepinones, dibenzoxepines and benzosuberones based p38α MAP kinase inhibitors: Their pharmacophore modelling, 3D-QSAR and docking studies. *Comput Biol Med* **110**, 175–185 (2019).

29.	Khedr, M. A., Mohafez, O. M. M. & Al-Haider, I. A. Virtual Screening-Based Discovery of Potent Hypoglycemic Agents: In Silico, Chemical Synthesis and Biological Study. *Curr Comput Aided Drug Des* **16**, 741–756 (2020).

30.	Makeneni, S., Thieker, D. F. & Woods, R. J. Applying Pose Clustering and MD Simulations To Eliminate False Positives in Molecular Docking. *J. Chem. Inf. Model.* **58**, 605–614 (2018).

31.	Malekipour, M. H., Shirani, F., Moradi, S. & Taherkhani, A. Cinnamic acid derivatives as potential matrix metalloproteinase-9 inhibitors: molecular docking and dynamics simulations. *Genomics Inform* **21**, e9 (2023).

32.	Masumi, M. *et al.* Methicillin-Resistant Staphylococcus aureus: Docking-Based Virtual Screening and Molecular Dynamics Simulations to Identify Potential Penicillin-Binding Protein 2a Inhibitors from Natural Flavonoids. *Int J Microbiol* **2022**, 9130700 (2022).

33.	Modi, P., Patel, S. & Chhabria, M. Structure-based design, synthesis and biological evaluation of a newer series of pyrazolo[1,5-a]pyrimidine analogues as potential anti-tubercular agents. *Bioorg Chem* **87**, 240–251 (2019).

34.	Moussa, N., Hassan, A. & Gharaghani, S. Pharmacophore model, docking, QSAR, and molecular dynamics simulation studies of substituted cyclic imides and herbal medicines as COX-2 inhibitors. *Heliyon* **7**, e06605 (2021).

35.	Muniz Seif, E. J., Icimoto, M. Y. & Silva Júnior, P. I. In silico bioprospecting of receptors associated with the mechanism of action of Rondonin, an antifungal peptide from spider Acanthoscurria rondoniae haemolymph. *In Silico Pharmacol* **12**, 55 (2024).

36.	Nagar, P. R., Gajjar, N. D. & Dhameliya, T. M. In search of SARS CoV-2 replication inhibitors: Virtual screening, molecular dynamics simulations and ADMET analysis. *J Mol Struct* **1246**, 131190 (2021).

37.	Omoboyowa, D. A. *et al.* Structure-based discovery of selective CYP17A1 inhibitors for Castration-resistant prostate cancer treatment. *Biol Methods Protoc* **7**, bpab026 (2022).

38.	Omoboyowa, D. A., Balogun, T. A., Omomule, O. M. & Saibu, O. A. Identification of Terpenoids From Abrus precatorius Against Parkinson’s Disease Proteins Using In Silico Approach. *Bioinform Biol Insights* **15**, 11779322211050757 (2021).

39.	Parcha, P. *et al.* Identification of natural inhibitors of Bcr-Abl for the treatment of chronic myeloid leukemia. *Chem Biol Drug Des* **90**, 596–608 (2017).

40.	Patel, H. M., Shaikh, M., Ahmad, I., Lokwani, D. & Surana, S. J. BREED based de novo hybridization approach: generating novel T790M/C797S-EGFR tyrosine kinase inhibitors to overcome the problem of mutation and resistance in non small cell lung cancer (NSCLC). *J Biomol Struct Dyn* **39**, 2838–2856 (2021).

41.	Patel, H. M., Ahmad, I., Pawara, R., Shaikh, M. & Surana, S. In silico search of triple mutant T790M/C797S allosteric inhibitors to conquer acquired resistance problem in non-small cell lung cancer (NSCLC): a combined approach of structure-based virtual screening and molecular dynamics simulation. *J Biomol Struct Dyn* **39**, 1491–1505 (2021).

42.	Pola, M., Rajulapati, S. B., Potla Durthi, C., Erva, R. R. & Bhatia, M. In silico modelling and molecular dynamics simulation studies on L-Asparaginase isolated from bacterial endophyte of Ocimum tenuiflorum. *Enzyme Microb Technol* **117**, 32–40 (2018).

43.	Rustamzadeh, A. *et al.* Targeting Caspases 3/6 and Cathepsins L/B May Decrease Laminopathy-Induced Apoptosis in Alzheimer’s Disease. *J Alzheimers Dis* **101**, 211–221 (2024).

44.	Shakibay Senobari, Z. *et al.* Chromone-embedded peptidomimetics and furopyrimidines as highly potent SARS-CoV-2 infection inhibitors: docking and MD simulation study. *BMC Res Notes* **16**, 224 (2023).

45.	Shrivastava, A. K., Kumar, S., Sahu, P. S. & Mahapatra, R. K. In silico identification and validation of a novel hypothetical protein in Cryptosporidium hominis and virtual screening of inhibitors as therapeutics. *Parasitol Res* **116**, 1533–1544 (2017).

46.	Sridhar, S. N. C., Palawat, S. & Paul, A. T. Design, synthesis, evaluation, and molecular modeling studies of indolyl oxoacetamides as potential pancreatic lipase inhibitors. *Arch Pharm (Weinheim)* **353**, e2000048 (2020).

47.	Suman, null, Chaudhary, M. & Nain, V. In silico identification and evaluation of Bacillus subtilis cold shock protein B (cspB)-like plant RNA chaperones. *J Biomol Struct Dyn* **39**, 841–850 (2021).

48.	Tania, M. *et al.* Cordycepin Downregulates Cdk-2 to Interfere with Cell Cycle and Increases Apoptosis by Generating ROS in Cervical Cancer Cells: in vitro and in silico Study. *Curr Cancer Drug Targets* **19**, 152–159 (2019).

49.	Tripuraneni, N. S. & Azam, M. A. A combination of pharmacophore modeling, atom-based 3D-QSAR, molecular docking and molecular dynamics simulation studies on PDE4 enzyme inhibitors. *J Biomol Struct Dyn* **34**, 2481–2492 (2016).

50.	Uba, A. I. & Yelekçi, K. Carboxylic acid derivatives display potential selectivity for human histone deacetylase 6: Structure-based virtual screening, molecular docking and dynamics simulation studies. *Comput Biol Chem* **75**, 131–142 (2018).

51.	Verma, A. K. *et al.* Identification of 1, 2, 4-Triazine and Its Derivatives Against Lanosterol 14-Demethylase (CYP51) Property of Candida albicans: Influence on the Development of New Antifungal Therapeutic Strategies. *Front Med Technol* **4**, 845322 (2022).

52.	Wan, Y. *et al.* In silico studies of diarylpyridine derivatives as novel HIV-1 NNRTIs using docking-based 3D-QSAR, molecular dynamics, and pharmacophore modeling approaches. *RSC Adv* **8**, 40529–40543 (2018).

53.	Zhao, X. *et al.* Synthesis and biological evaluation of 6-hydroxyl C-aryl glucoside derivatives as novel sodium glucose co-transporter 2 (SGLT2) inhibitors. *Bioorg Med Chem Lett* **28**, 2201–2205 (2018).

54.	Behera, B. K. *et al.* Molecular characterization and structural dynamics of Aquaporin1 from walking catfish in lipid bilayers. *Int J Biol Macromol* **196**, 86–97 (2022).
